# Multimodal Biophysical Analysis of Morpho-Phenotypic and Morpho-Functional Dynamics in Normal and Cancer Spheroids within Engineered Microenvironments

**DOI:** 10.1021/cbmi.5c00255

**Published:** 2026-04-29

**Authors:** Srijani Dey, Shreya Adhikary, Priyanka Das, Pallab Datta, Subhadip Chakraborty, Ananya Barui

**Affiliations:** 1 Centre for Healthcare Science and Technology, 30130 Indian Institute of Engineering Science and Technology, Shibpur, Howrah 711103, India; 2 Department of Pharmaceutics, 231950 National Institute of Pharmaceutical Education and Research, Kolkata 700054, India; 3 Institute of Engineering and Management, Management House, D-1, Sector-V, Saltlake Electronics Complex, Kolkata, West Bengal 700091, India; 4 University of Engineering and Management, New Town University, Area, Plot no. III, B/5, New Town Rd, Action Area III, Newtown, Kolkata, West Bengal 700160, India

**Keywords:** spheroids, heterogeneity, morphology, autofluorescence, impedance

## Abstract

Three-dimensional spheroid models provide physiologically relevant systems to investigate cancer progression and stem cell dynamics. However, most existing literature focuses primarily on morphological or biochemical characterization, limiting a comprehensive understanding of the coupled biophysical, morphological, and molecular dynamics of spheroids within engineered microenvironments. Addressing this gap, the present study develops a multimodal biophysical framework to comprehensively assess the morpho-phenotypic, metabolic, and electrochemical characteristics of spheroids. Here, we report a comparative analysis of spheroids derived from mesenchymal stem cells (MSC), a mixed population of cancer stem cells and cancer cells (A549), and differentiated cancer cells (MCF-7), cultured in an ECM-mimetic alginate-gelatin hydrogel. Relevant morpho-phenotypic parameters were analyzed at 3, 7, and 14 days from confocal images and cytoskeletal mapping, revealing distinct trends in size, circularity, solidity, and entropy. All spheroids exhibited time-dependent growth (40–60 to 120–240 μm), with cancer spheroids (A549 and MCF-7) showing significantly greater volumetric expansion. Day 7 emerges as a critical transition point marked by an alteration in the morphological trend. A549 spheroids displayed higher entropy, reflecting increased heterogeneity. Autofluorescence spectroscopy revealed distinct redox dynamics of spheroids from different origins. Cancer spheroids showed enhanced NADH, FAD, and porphyrin signatures reflecting redox alterations and Warburg-like effects, wherein MSC spheroids showed a controlled shift toward oxidative phosphorylation. The impedance spectroscopy further distinguished spheroids through capacitance–resistivity relationships. Notably, A549 spheroids exhibit dynamic transitions between stem-like and malignant states, consistent with their intrinsic heterogeneity. Overall, this integrated approach correlates growth, redox status, and dielectric behavior, offering a translational platform for drug screening, disease modeling, and regenerative medicine.

## Graphical abstract

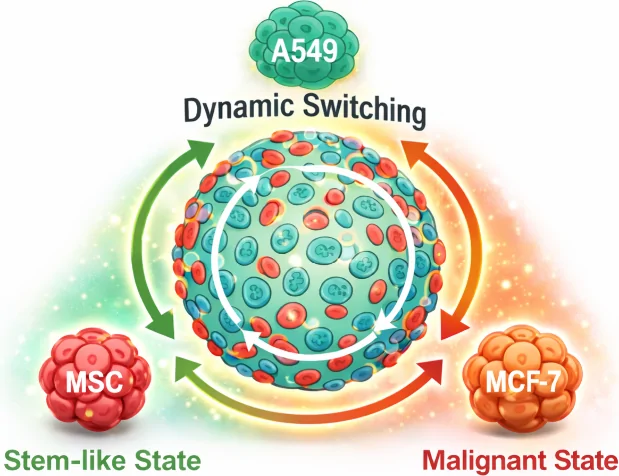

## Introduction

The connection between tissue regeneration and cancer is well recognized. However, the biological intricacies encompassing cellular interactions, metabolic patterns, and biophysical aspects exhibit context-dependent variation, underscoring the need for a systemic investigation. Although significant progress has been made in studying cancer using three-dimensional (3D) culture models, the structural and functional dynamics of cancer cells compared with undifferentiated cells or cancer stem cells remain unexplored. In this regard, spheroids, also known as “microtissues”, provide an excellent 3D system that can mimic the interactions of various cell types owing to their superiority over conventional monolayer cultures.^1,2^ Spheroids also provide a suitable platform for comparing the interactions and heterogeneity of various cell sources, including stem cells, cancer stem cells, and cancer cells. The structure, activity, and propulsion systems of cells in such spheroids shed light on the molecular underpinnings of regeneration versus malignant transformation.^3^

The development of a 3D engineered microenvironment using spheroid models is important for investigating the morpho-phenotypic or morpho-functional dynamics of cells from distinct origins.^4–7^ Studies have shown that extracellular matrices (ECMs) derived from hydrogel can accurately control spheroid size and morphology.^8^ Cellular morphology reflects the functional state of different cell types. Several studies compared the transcriptomic profiles of spheroids with *in vivo* outcomes, which sometimes contradict the fidelity of spheroids as a viable alternative to conventional 3D models.^8–10^ At present, few studies have interlinked the morphological features of spheroids to their transcriptomic profiles.^11^ Cheng et al. have shown in their study that the morphology of multicellular tumor spheroids (MCTs) is modulated by cell type, cell density, culture conditions, and mechanical stress.^48^ Interestingly, multiple features of spheroid morphology (such as spheroid size and the number of nuclei) often change together and describe spheroids unanimously. However, the uncorrelated features introduce a new dimension of the spheroid morphology, possibly distinct for specific cell types. Altogether, different morphological features like shape, size, orientation angle, solidity, and eccentricity are emerging readouts to underpin the molecular profiling of spheroids.^8,13–17^

In addition to morphology, biochemical and electrochemical profiles play an important role in understanding cellular heterogeneity under both cancerous and naïve conditions. Characteristics such as cell–cell cohesion, intercellular interaction, and metabolite production by spheroids can provide insights into their physiological status. Quantification of these parameters using emerging technologies like autofluorescence and impedance spectroscopy can therefore establish spheroids as potential 3D platforms for early disease diagnosis and the development of personalized and precision medicine.^18–21^ Autofluorescence-based studies indicate the metabolic activities of various spheroids and their interactions with the immediate microenvironment.^20–22^ The autofluorescence signatures of normal and cancer spheroids reflect the accumulation and alterations of different endogenous metabolites like NADH, FAD, and porphyrin and reflect the morpho-functional dynamics of cells.^20,21,23^ Quantification of these metabolites using fluorescence images of dense three-dimensional spheroids facilitates understanding of the internal metabolic information. It also tracks the development of complex spheroid architecture.^24^ However, external fluorescent dyes can permeate the cell membranes, and their diffusion through the surrounding hydrogel matrix often exhibits strong nonspecific expressions.^22,25,26^ In this regard, cellular autofluorescence imaging serves as a powerful alternative that captures the exact metabolic status of cells without imposing toxicity on living systems. Cellular energy metabolism is particularly important for understanding cellular status in physiological and pathological situations. Perturbation in energy metabolism is a crucial indicator of pathogenesis and could be used as a potential theranostic target.^27^

In parallel to biochemical signatures, electrochemical communication among cells plays a crucial role in modulating intrinsic cellular behavior, although this phenomenon remains relatively underappreciated.^27^ Recent studies suggest that bioelectrical interactions hold immense potential for understanding molecular mechanisms underlying both tissue regeneration and malignancy.^28^ In spheroids, increases in biomass and cell density have been shown to influence bioelectrical properties, particularly by increasing impedance.^29–32^ The impedance spectra of cancer spheroids differ significantly from those of undifferentiated or normal cells, largely due to the heterogeneous cell population and abnormal differentiation driven by the cancerous microenvironment.^33–36^ This is primarily attributed to protein–ion interactions, which alter the distribution of free charges and thereby affect the intracellular conductivity.^29,31^ Consequently, the interaction of spheroids with their surrounding microenvironment, along with the resulting cellular behavior, has been investigated recently using electrochemical impedance spectroscopy. Researchers have utilized bioelectrical measurements to monitor tumor spheroid dynamics in real time and have applied them to assess the correlations between electrical properties and spheroid morphology, including shape and size.^32,37^ Additionally, impedance has been employed to evaluate the cytotoxicity of spheroids under treatment with different drugs. Importantly, combining autofluorescence with impedance spectroscopy enables functional imaging, offering an integrated view of metabolic and electrochemical dynamics.^27^

Considering the aforementioned, this study aims to understand the morpho-phenotypic and morpho-functional dynamics of spheroids from three different cell lines—MSCs (mesenchymal stem cells), A549 (lung adenocarcinoma), and MCF-7 (breast adenocarcinoma). MSCs are selected as a nonmalignant cell type, while A549 and MCF-7 cells are used as malignant models, as they are clinically relevant epithelial tumor cell lines. To understand the characteristic differences or similarities among cells in distinct microenvironments provided by spheroids, these models are used. Although tissue-specific metabolic discrepancies are recognized, the current manuscript aims for a relative, microenvironment-mediated perspective on structural and functional dynamics. The inclusion of cancer models from different tissue lineages is able to strengthen the translational relevance of the platform by demonstrating its applicability across diverse malignancies. The morpho-phenotypic characteristics of various spheroids at different culture times are analyzed from confocal 3D-mapped images. Furthermore, the autofluorescence characteristics of normal vs cancer spheroids grown within alginate-gelatin hydrogel droplets are assessed to evaluate the metabolic and redox reactions. Impedance is subsequently utilized to understand the interaction of spheroids derived from different cell types with their bioengineered microenvironment.

The existing literatures highlight the need for a comprehensive study of the structural, metabolic, and electrochemical dynamics of spheroids within engineered microenvironments. By combining morpho-phenotypic analysis, autofluorescence-based metabolic profiling, and electrochemical assessment, the present study aims to provide an integrated framework to comprehend the functional heterogeneity of spheroids derived from diverse cellular origins. Such a multimodal strategy may facilitate the development of robust 3D platforms for studying cancer progression, personalized screening, and translational biomedical applications.

## Experimental Section

### Materials

Cell lines A549 (lung adenocarcinoma cells), MSC (mesenchymal stem cells), and MCF-7 (breast adenocarcinoma) were generously gifted. Roswell Park Memorial Institute (RPMI with phenol red), Dulbecco’s modified Eagle’s medium high-glucose (DMEM high glucose, with phenol red), Alpha modified Eagle’s medium (αMEM), fetal bovine serum (FBS), penicillin/streptomycin, and trypsin/EDTA were obtained from Gibco (Carlsbad, California, USA). Sodium alginate, gelatin, calcium chloride (CaCl_2_), paraformaldehyde, phalloidin, and DAPI were purchased from Sigma-Aldrich (US). Triton X-100 was purchased from Sisco Research Laboratories (SRL), India. Calcein AM and propidium iodide were purchased from HiMedia (US). Syringe filters with 0.2 μm pore size were acquired from Sartorius (Germany). For the preparation of buffers and samples used in experiments, deionized water was used.

### Cell Culture

A549 cells were cultured in RPMI media supplemented with 10% FBS, 1% penicillin–streptomycin, and 1% amphotericin B in a humidified atmosphere at 37 °C and 5% CO_2_. DMEM high-glucose and αMEM were used to culture MSC and MCF-7 cells, respectively, under similar culture conditions. After reaching ∼70% confluency, cells were detached from the flask using 0.25% trypsin. A hemocytometer was used to count cells before mixing in alginate-gelatin hydrogel.

### Development of Spheroids

1% (w/v) sodium alginate dissolved in PBS buffer (pH 7.4) was autoclaved, mixed with 0.5% (w/v) gelatin solution, and then passed through a 0.22 μm syringe filter. 200 μL of suspension of each cell type (10^6^ cells mL^–1^) was mixed separately with alginate-gelatin hydrogel solution (pH 7.4). 1 mL of cell hydrogel was loaded into the syringe and dropped into a 100 mM CaCl_2_ solution. The spheroids were cultured for 3, 7, and 14 days.

### Morphological Assessment of Spheroids

The morphologies of spheroids cultured in alginate-gelatin hydrogel droplets were inspected at 3, 7, and 14 days^38,39^ and were assessed from the images acquired through a differential interference contrast (DIC) microscope (Leica Stellaris 5, Germany). A truncated 1000 μL pipet was used to prevent shear on the spheroids during transfer to the coverslip, and the spheroids were fixed with 4% paraformaldehyde (PFA) for 15 min at room temperature (RT). Following fixation, spheroids were washed three times with PBS and imaged at 10× magnification under DIC mode (Leica Stellaris 5, Germany).

Morphological parameters, including cellular characteristics such as area, volume, diameter, solidity, circularity, eccentricity, major and minor axis lengths, aspect ratio, orientation angle, entropy, and relative intensity, were extracted from DIC images for characterizing the classes. The parameters were calculated using the following formulas:

Area of the spheroids: The entire surface area of the spheroids is known as the cellular area and represented in eq 1

$$A=\underset{\left(x,y\right)\in R}{\sum }f\left(x,y\right)$$ 1

where *R* is the set of pixels present in spheroids. The spheroid image is represented as *f*(*x*,*y*).

Volume of the spheroids: The voxel count within the 3D segmented spheroid region and represented in eq 2

$$\begin{array}{c} V=\underset{\left(x,y,z\right)\in R}{\sum }f\left(x,y,z\right) \end{array}$$ 2

Diameter of the spheroids: The diameter of the spheroids. It is represented in eq 3.

$$\begin{array}{c} D=\sqrt{\frac{4A}{\pi }} \end{array}$$ 3

Solidity of spheroids: Ratio of the area of the spheroid to the area of its convex hull. It is represented in eq 4

$$\begin{array}{c} S=\frac{A}{A_{convex}} \end{array}$$ 4

Circularity of spheroids:

The measurement of the circle perfectly fitted to the spheroid. It is represented in eq 5.

$$\begin{array}{c} C=\frac{4\pi A}{P^{2}} \end{array}$$ 5

where *P* is the perimeter of the spheroids.

Eccentricity of the spheroids: The ratio of the distance between foci of the ellipse fitted to the spheroid to its major axis length. It is represented in eq 6 as

$$\begin{array}{c} E=\frac{\sqrt{c^{2}-d^{2}}}{c} \end{array}$$ 6

where *c* is the major axis length and *d* is the minor axis length of the spheroid.

Major and minor axes and aspect ratio of spheroids: The longest and shortest axes of the ellipse fitted to the spheroids are known as the major and minor axes, respectively. The ratio of the major to minor axis lengths of a spheroid is its aspect ratio.

Orientation angle of spheroids: The angle between the major axis of the spheroids fitted ellipse, and the *x*-axis is known as the orientation angle.

Relative intensity of spheroids: The average intensity inside the spheroid compared to the background is the relative intensity of spheroids and is expressed in eq 7

$$\begin{array}{c} RI=\frac{\mu_{spheroid}}{\mu_{background}} \end{array}$$ 7

where μ_spheroids_ is the mean intensity of the spheroids and μ_background_ is the mean background intensity except for spheroids.

Entropy is the statistical measurement of randomness used to characterize the texture of spheroid images. Entropy is calculated using the formula represented in eq 8.

$$\begin{array}{c} Entropy=-sum\left(a.\times \log\;2\left(a\right)\right) \end{array}$$ 8

where *a* contains normalized histogram counts.

### SEM Study

For SEM, spheroids embedded in hydrogel were collected using wide-mouth tips, fixed with 2.5% glutaraldehyde (pH 7.4) on a coverslip, and incubated for 1 h. After removing excess solution, spheroids were subjected to serial dehydration with 10–100% ethanol after every 10 min. The spheroids were washed with methanol and air-dried. Samples were subjected to gold spluttering for 2 min, followed by image acquisition at 5 kV with a magnification of 5000× (Zeiss Gemini SEM, USA).

### Live/Dead Cell Staining

For the live/dead cell assay, harvested spheroids at different time points were stained with Calcein AM and propidium iodide. The culture media were removed, washed thoroughly, and incubated with 100 μL of staining solution (5 μL of 2 μM Calcein AM and 20 μL of 4 μM propidium iodide (PI) diluted in 10 mL of PBS) in the dark for 30 min. The spheroids were imaged using 488 nm excitation for Calcein AM and 561 nm excitation for PI. The confocal microscope (Leica Stellaris 5, Germany) was used to acquire images at 10× magnification.

### Immunofluorescence Staining and 3D Mapping of Spheroids

Spheroids were transferred to a coverslip, washed thoroughly with PBS at pH 7.4 for 5 min, and fixed with 4% paraformaldehyde solution for 15 min at RT. Spheroids were permeabilized using 0.2% Triton X-100 for 1 h in a shaker. This was followed by 30 min of incubation with Alexa Fluor 647 phalloidin and 15 min of incubation with 2–5 μL of DAPI (4′,6-diamidino-2-phenylindole), followed by a thorough wash with PBS. The confocal microscope (Leica Stellaris 5, Germany) was used to acquire images at 10× and 63×/1.2 water objectives, with excitation at 561 and 405 nm and emission at 650 and 488 nm. The scanning speed is set at 100 scans per sample. The experiment was repeated every 3, 7, and 14 days.

During imaging, when spheroids in the alginate-gelatin hydrogel are focused on a particular layer, images of spheroids in other layers are often blurred. Hence, the scanning time, number of scans, and step size were optimized to image the 3D map of spheroids. The step size was kept at 1 μm, with a scanning speed of 400 scans per sample, and the scanning time was 10 to 15 min. The Leica Application Suite X (Las X) was used to measure the spheroid dimensions in the Z plane (height) and the ZY orthogonal view (distance between the lowest and highest points of the spheroid).

### Analysis of Metabolic Parameters

To assess the intrinsic autofluorescence, fixed spheroids were imaged at 10× magnification. The spheroids were excited using a confocal laser scanning microscope (Leica Stellaris 5, Germany) at 405, 488, 514, 561, and 638 nm. The emission spectra were recorded between 410 and 480, 490–510, 520–560, 565–620, and 640–750 nm. Laser power was optimized to 50%, and 13 scans were taken for each wavelength. Experiments were performed in triplicate.

Redox ratio is considered a measurement tool for cellular metabolism. The redox ratio was calculated from normalized autofluorescence data for detected NADH and FAD. The reduction and oxidation ratio was computed using eq 9.

$$\mathrm{Redox}\;\mathrm{ratio}=[{\mathrm{FAD}}_{\mathrm{intensity}}/\left({\mathrm{FAD}}_{\mathrm{intensity}}+{\mathrm{NADH}}_{\mathrm{intensity}}\right]$$ 9

where FAD and NADH are emission intensities at ∼510 and 450 nm, respectively.

### Impedance Measurement

The electrochemical impedance of spheroids was measured using an Electrochemical Workstation (Metrohm, Germany). The hydrogel droplets containing spheroids were placed on screen-printed electrodes (SPE) (Zensor, TE100) connected to the workstation. A 250 mV AC was applied at frequencies (10 Hz to 1 MHz). The experiment was performed in triplicate. The graphs were plotted with Origin 2019b. The visual representation of the experimental setup with the sample placed on the electrode is illustrated in Figure S1.

### Statistical Analysis

All experimental data were obtained in triplicate, and values were reported as the mean ± SD or mean ± 95% confidence interval. GraphPad Prism 9.0.0 (GraphPad Software, San Diego, California, USA) was used to perform statistical analysis. An ordinary one-way ANOVA or a two-way ANOVA was used to assess statistical significance among the six experimental groups, with the Tukey test used for pairwise comparisons of means. The significance level was set at *p* < 0.05. A Gaussian fit (Origin 2019b) was performed to normalize the spectral data obtained from the confocal microscope (Table S1).

## Results and Discussion

### Morpho-Phenotypic Characterization of Normal and Cancer Spheroids at Different Maturation

To investigate spheroid growth dynamics, morphological changes are analyzed over time from DIC, DAPI-stained, and 3D reconstructed images acquired at different time points. This analysis facilitates in a comprehensive assessment of morphological alterations and spatial arrangement across different spheroids derived from different cell types. Figure 1 represents the DIC (Figure 1A), DAPI (Figure 1B), and 3D mapping (Figure 1C) images of spheroids at various time points. The spheroids on day 3 across all groups are in the 40–60 μm size range. By day 14, the sizes significantly increase (*p* < 0.05) to the range of 120–240 μm (Figure 1C). MSC spheroids show a significant (*p* < 0.05) increase in size from 40 μm at day 3 to 180 μm at day 14. However, A549 and MCF-7 spheroids exhibit a significant (*p* < 0.05) increase in size from 60 μm at day 3 to 200 and 250 μm, respectively, at day 14. The significant (*p* < 0.05) increase in size of MCF-7 and A549 spheroids at day 14 compared to MSC spheroids indicates the higher proliferative rate in cancer cells.^40,41^ On the other hand, MSC exhibits a moderate increase in spheroid size, indicating regulated growth kinetics.

**Figure 1:**
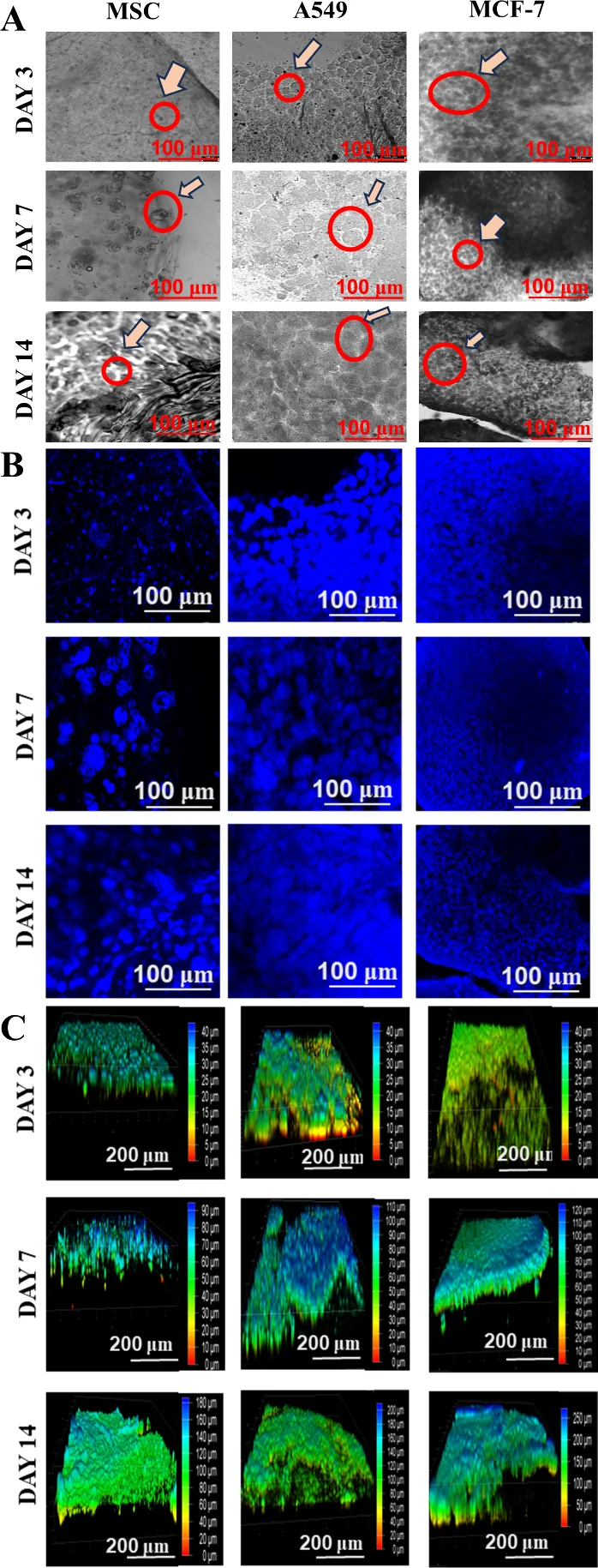
Morphological assessment of MSC, A549, and MCF-7 spheroids at 3, 7, and 14 days is performed from the (A) DIC images. Red circles indicate the presence of spheroids in the alginate-gelatin hydrogel. (B) DAPI staining of the spheroids and (C) 3D mapping of the spheroids, indicating their arrangement across different layers, spatial distribution, and varying sizes. All the images are taken at 10× magnification.

Quantitative changes in spheroid morphology are further assessed at different time points based on several parameters such as area, volume, size, eccentricity, and orientation angle. Diameter and area represent spheroid proliferation, wherein circularity and solidity indicate the cell–cell adhesion and invasive boundary behavior of the spheroids, respectively. Aspect ratio and eccentricity confirm the changes in symmetry across the different spheroid morphologies during growth.^42^

Spheroids derived from MSC, A549, and MCF-7 (1.4 × 10^4^ μm^2^) cells depict a similar trend in area for all time points (Figure 2A). On day 7, the lowest area is observed across all spheroids at approximately 7.7 × 10^4^ μm^2^, which increases significantly to 10 × 10^4^ μm^2^ across all spheroids on day 14 (Figure 2A). No significant change in the diameters of A549 and MCF-7 spheroids was observed between day 3 and day 14 (125 and 127 μm, respectively); however, a significant (*p* < 0.0001) decrease in diameter is observed on day 7. The MSC spheroids show a slightly smaller diameter than other spheroids at all time points, and, similar to other groups, a significant increase (*p* < 0.001) is observed from days 3 to 14 (Figure 2B). The increase in spheroid diameter at 14 days in all study groups indicates higher cellular proliferation.

**Figure 2:**
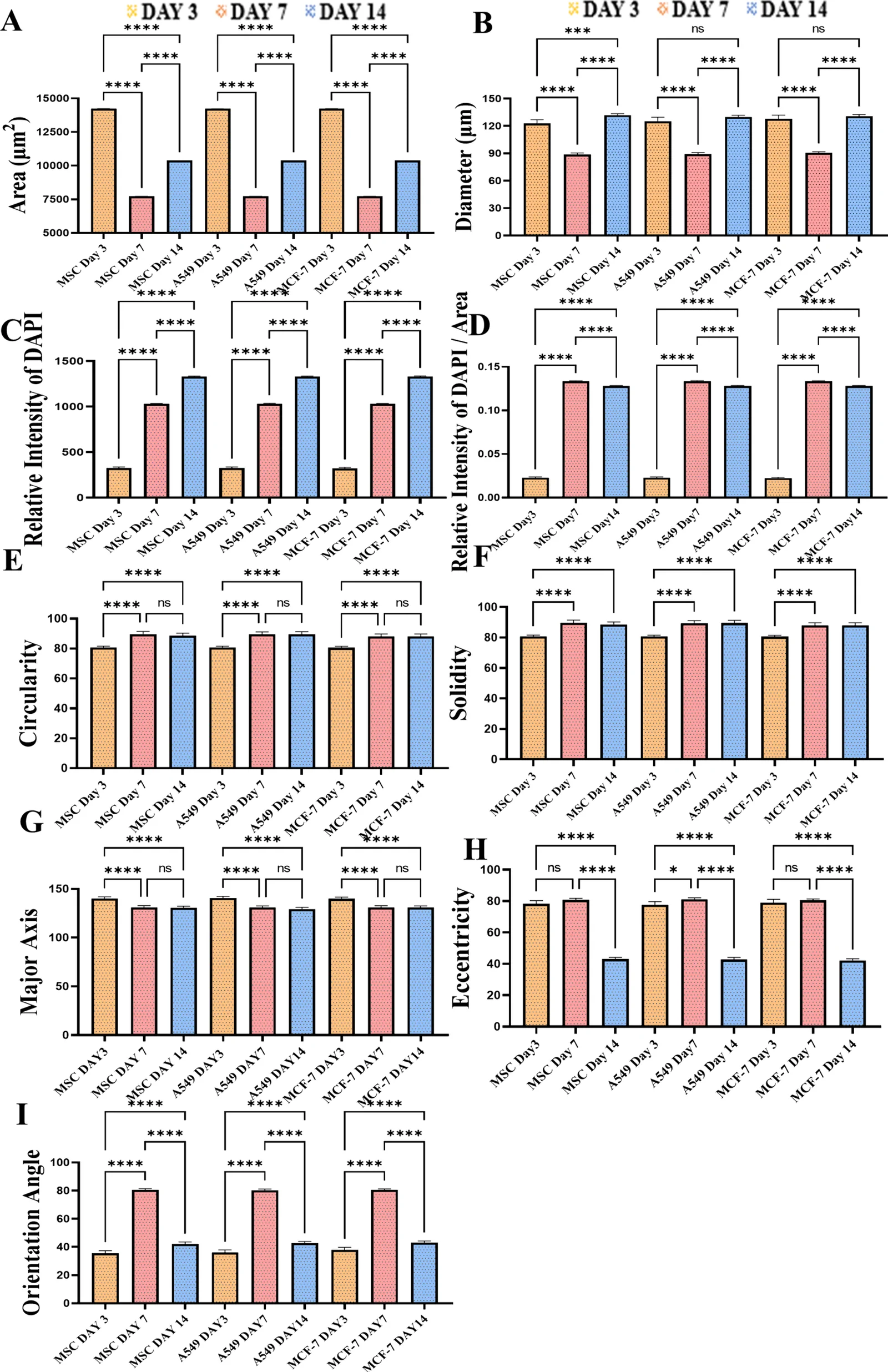
The graphical representation of the morphological parameters extracted from the confocal images of the MSC, A549, and MCF-7 spheroids at different time points. (A) Area, (B) diameter, and (C) relative intensity of the spheroids stained with DAPI. (D) Relative intensity of DAPI/area; (E) circularity; (F) solidity; (G) major axis; (H) eccentricity; and (I) orientation angle. The data in (A–I) are presented as mean ± 95% confidence interval, *n* = 3. Significant difference between spheroids and days is indicated as follows: ****p < 0.0001, ***p < 0.001, **p < 0.01, and *p < 0.05.

The confocal images show that DAPI intensity (Figure 1B) is visibly higher across all spheroids with increasing time. Furthermore, the relative intensity is calculated and represented in Figure 2C, which also indicates a significant (*p* < 0.0001) increase in intensity for all spheroids from days 3 to 14. At day 14, the intense expression of DAPI reflects higher cellular proliferation in spheroids. Interestingly, the area-to-relative-intensity ratio increases on day 7 and decreases on day 14 for all spheroids (Figure 2D), suggesting a shift in growth dynamics during maturation, as observed in the previous 3D volumetric reconstruction (Figure 1C). Although projected morphometric parameters such as area and diameter show a significant decrease (*p* < 0.0001) on day 7, 3D volumetric reconstruction (Figure 1C) and nuclear intensity (Figure 2C) indicate continued biological growth. This typical phenomenon is due to an initial increase in the size driven by rapid proliferation, followed by the formation of a compact structure at a later stage of development.^43^ During this phase, enhanced cell–cell adhesion and structural reorganization increase cell density while reducing the lateral spreading, projected area, and diameter. The increase in circularity and solidity also supports this phenomenon.^14,15^ Circularity is primarily used to characterize the surface structure. The circularity in the range of 85–100 indicates that, over time, the spheroids have attained a spherical structure. All three different spheroids MSC, A549, and MCF-7 have demonstrated a significant increase (*p* < 0.0001) in the circularity from 70 on day 3 to 90 on day 14. Solidity helps in understanding the compactness of spheroids. All the spheroids show a significant (*p* < 0.0001) increase in circularity, solidity, and major axis until day 7, and no significant change is observed at day 14 (Figure 2E–G).

The spheroids developed in hydrogel demonstrated heterogeneity. Typically, cancer spheroids demonstrate tumor clustering in specific regions; in contrast, MSC spheroids show a more even distribution in the hydrogel (Figure 1C). On day 3, the eccentricity of spheroids shows a value of around 80–90, which suggests the irregularity of the spheroids (Figure 2H). MSC and MCF-7 spheroids show no significant change in eccentricity from day 3 to day 7, while A549 shows a significant increase (*p* < 0.05). However, on day 14, a significant (*p* < 0.0001) decrease in eccentricity across all spheroids is observed, indicating the progressive acquisition of characteristic shapes of spheroids over time. The orientation angle across all spheroids significantly (*p* < 0.0001) increases to 80° on day 7 and further decreases (*p* < 0.0001) to 42–47° (Figure 2I) on day 14, while changes in eccentricity and the major axis length reflect changes in spheroid orientation. The orientation angle quantifies the degree of these changes. These parameters help in understanding the development of minute morphological changes in different spheroids at different time points.

The live and dead assay (Figure 3A) is performed to assess the viability of cells in spheroids at different time points. Morphometric and viability analyses show a moderate correlation between spheroid growth kinetics and cellular survival patterns. MSC spheroids maintained higher viability on day 3, as their smaller size facilitates efficient nutrient diffusion compared to cancer spheroids.^44^ On day 7, A549 spheroids show (*p* < 0.05) significantly higher live cell intensities (Figure 3B). In contrast, both MSC and MCF-7 cells show an increasing number of dead cells (*p* < 0.05) at the same time point (Figure 3B). This behavior may be related to structural compaction and the increased solidity at day 7 (Figure 2F), indicating a higher cellular density that restricts oxygen and nutrient diffusion, thereby promoting hypoxia-induced necrotic core formation.^43,45^ On the contrary, A549 spheroids maintained significantly higher viability during day 7, which possibly indicates the highly aggressive nature of these cells that tend to form irregular spheroids, which is evident in Figure 3C.^46^ However, in the later stage of development (day 14), viability across all spheroids has improved despite increased size, indicating architectural stabilization and metabolic adaptation that confined necrosis to the core while maintaining viable, proliferative outer layers.^47^

**Figure 3:**
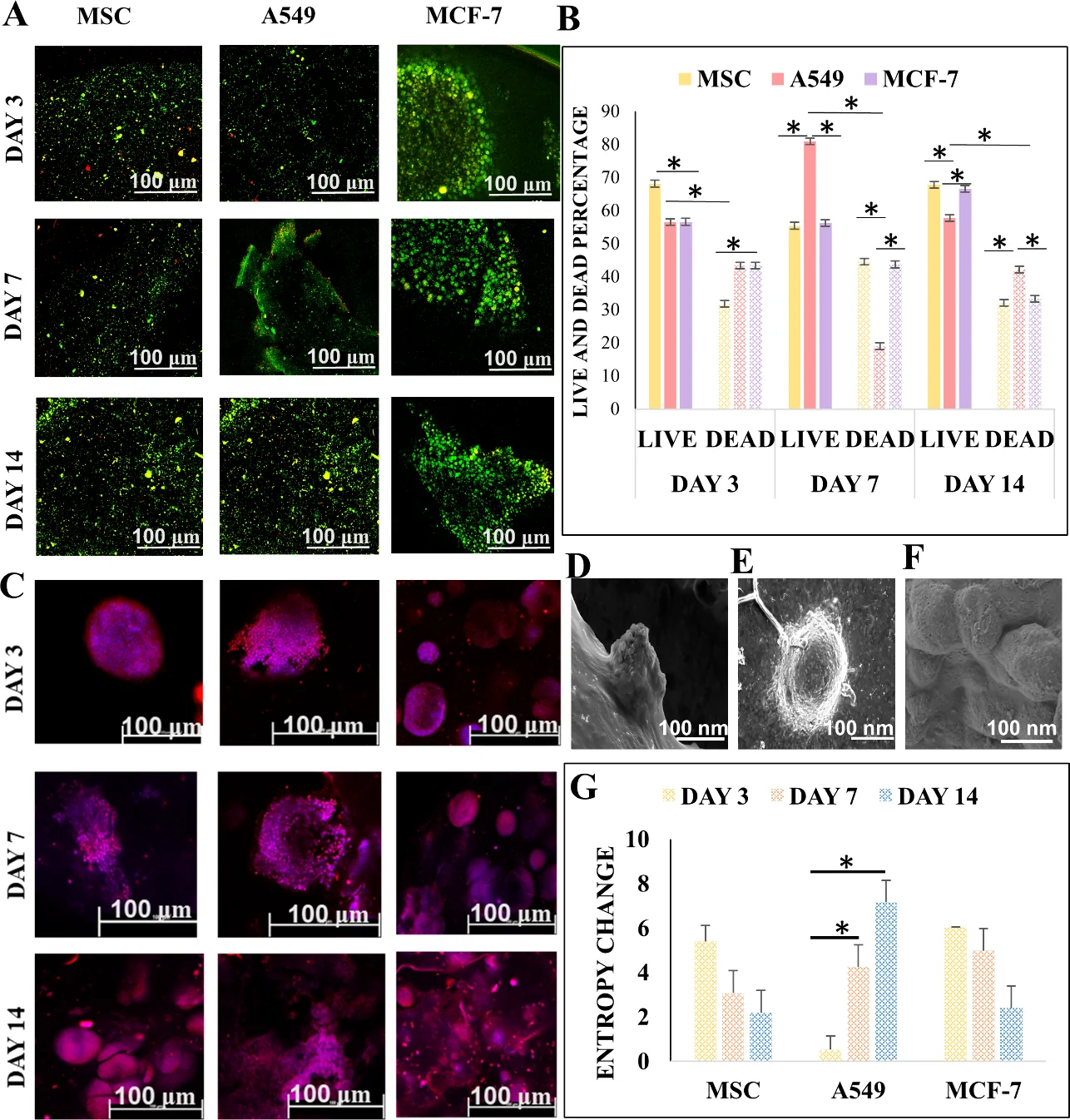
Depicts the growth and differentiation of different types of spheroids. (A) cellular viability of different spheroids grown in alginate-gelatin hydrogel at days 3,7, and 14; (B) Graphical representation of the % of live and dead cells in spheroids at 3,7, and 14 days; (C) Rhodamine- phalloidin staining of different spheroids images (63X) shows the cytoskeleton alteration at different time points (3, 7, and 14 days); the ultrastructure of (D) MSC, (E) A549, and (F) MCF-7 spheroids grown at day 7 (scale: 10 nm). (G) Graphical representation of entropy of different spheroids. All experiments were performed in triplicate. Significant difference is set at *p < 0.05.

Generally, as spheroids mature, their shapes change over time, as evident in the previous results. To understand the shape and distribution of actin in the spheroids, rhodamine-phalloidin staining is performed, and the results are shown in Figure 3C.^13,48^ The distribution of the cytoskeleton provides crucial information about cell–cell interactions among different spheroids and the minute morphological changes occurring during spheroid maturation. The microstructures of MSC and MCF-7 show spherical, regular structures at all time points; however, A549 shows irregular shapes. A549 cells exhibit a rugged morphology with numerous bleb-like structures, which is further confirmed by the ultrastructure images (Figure 3E). The SEM image of A549 shows cellular projections, and these protrusions are critical cell-surface structures that connect the cells in 3D tumor environment via the actin cytoskeleton.^49^ Bleb formation is also observed in A549 spheroids, which is a morphological feature associated with metastatic and aggressive cancers.^46,50^ These blebs are found to establish prompt septin signals, substituting for the loss of anchorage-dependent signaling upon substrate detachment. Once this signaling is established, cancer cells can travel through low-adhesion environments. MCF-7 depicts an ellipsoidal structure in the earlier stage and develops into a grapelike tubular structure in the later stage (Figure 3C), which is also confirmed from the ultrastructure imaging of MCF-7 (Figure 3F). MSC-derived spheroid shows a lobbed-like structure (Figure 3D). At day 7, MSC spheroids are more spherical, but at the later stage, the MSC spheroids attained a cluster of ellipsoidal structure (Figure 3C).^51,52^

To understand the spheroid dynamics, the entropy is calculated.^15–17^ A549 spheroids show the highest entropy value at the later stage (day 14), which is a significant (*p* < 0.05) increase from day 3 to day 14. The increased entropy value indicates the aggressive behavior of the A549 spheroids,^53^ while MCF-7 and MSC initially show higher entropy values that decrease over time, indicating that the spheroids are becoming more compact (Figure 3G).

Taken together, the morpho-phenotypic characterization indicates the distinct behaviors among MSC, A549, and MCF-7 spheroids. MSC spheroids exhibit regulated growth, an organized cytoskeletal architecture, and relatively stable morphology, whereas rapid expansion, morphological irregularities, and higher entropy in cancer spheroids indicate their aggressive nature. This section demonstrates that the studied parameters collectively reflect the intrinsic differences between the regenerative and cancerous cellular systems during maturation.

### Morpho-Functional Characterization of Spheroids from Autofluorescence Imaging

To further correlate the morphological alterations with the viability of spheroids, cellular metabolic dynamics are investigated through autofluorescence imaging. Spheroids are excited at different wavelengths of light, and endogenous fluorescence emission is measured to understand the intensities of different metabolites (Figure 4A–C) like NADH, FAD, and porphyrin. From the normalized spectral data of metabolites (Figure 4E), A549 spheroids show the highest intensities of NADH and FAD at day 3 (Figure 4E). At day 7, the MSC spheroids show a significant increase in FAD intensity (*p* < 0.05) while MCF-7 spheroids show a significant (*p* < 0.05) increase in NADH expression. However, at day 14, the A549 spheroid shows the highest NADH production, while the MCF-7 shows a significant (*p* < 0.05) increase in FAD production.

**Figure 4:**
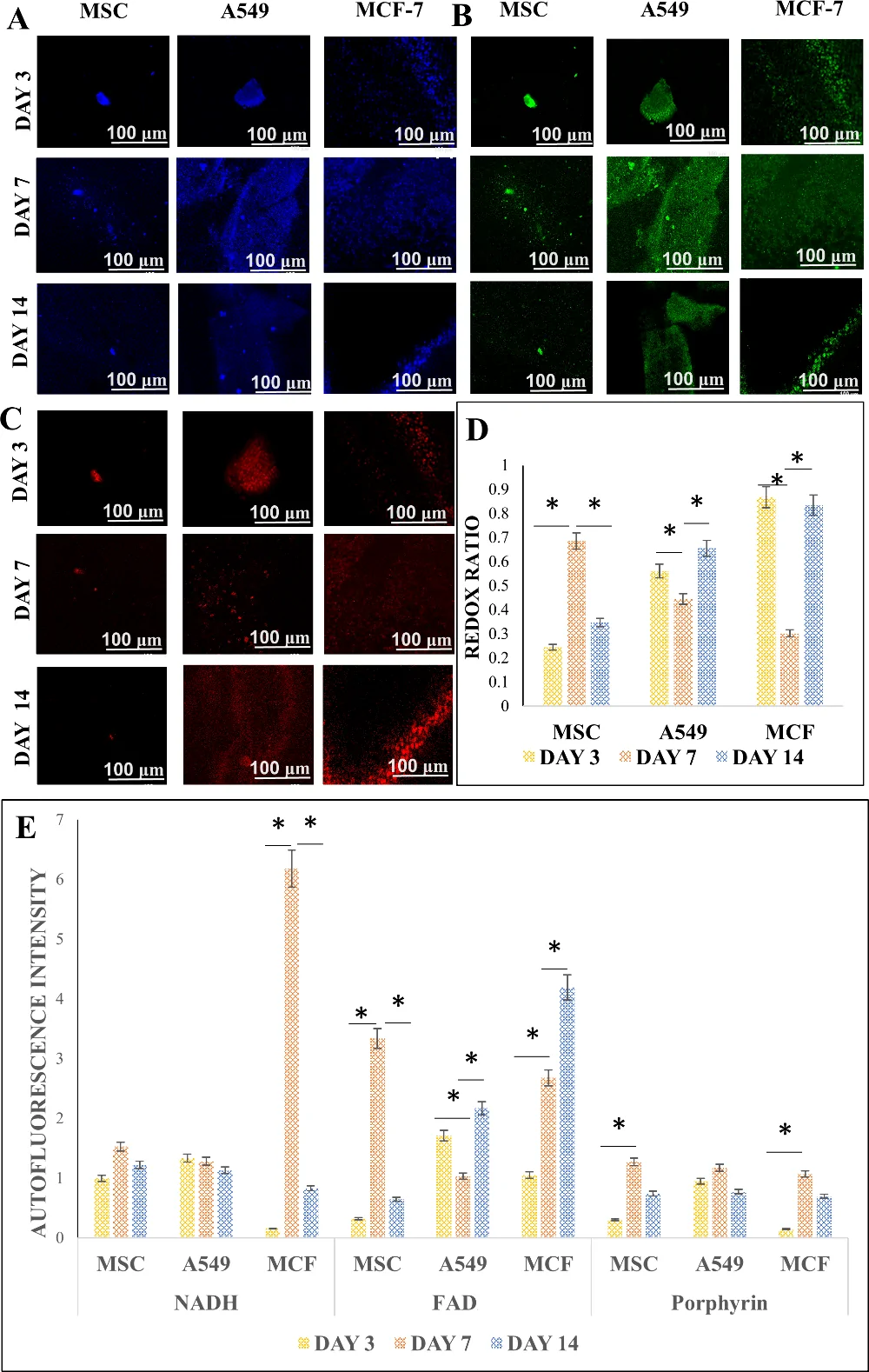
Autofluorescence characteristics of different spheroids at 3, 7, and 14 days. Spheroids were imaged at the (A) blue channel (λ_excitation_ at 405 nm) for detecting the presence of NADH; (B) green channel (λ_excitation_ at 488 nm) for detecting the presence of FAD, and (C) red channel (λ_excitation_ at 550 nm) for detecting the presence of porphyrin. Graphical representation of the (D) redox ratio of spheroids and (E) normalized autofluorescence intensities of spheroids at 3, 7, and 14 days. The data in (D, E) are presented as mean ± SD; *n* = 3; *p < 0.05.

The redox ratio (Figure 4D) is calculated from the metabolites’ intensities, which provide information about cellular metabolism. On day 3, MCF-7 spheroids depict a significantly (*p* < 0.05) higher redox ratio of 0.8, wherein the least redox ratio of 0.2 is observed in MSC spheroids, which is possibly associated with an increased rate in glycolysis.^54^ However, a significant (*p* < 0.05) increase in redox ratio on day 7 is observed for MSC spheroids. This phenomenon reflects the shift of metabolism from glycolysis to the TCA cycle, which is generally observed during the maturation of MSC.^55^ On day 14, there has been a significant (*p* < 0.05) increase in the redox ratio in MCF-7 and A549 spheroids compared to the MSC spheroids. Cancer spheroids exhibit increased "Warburg effects"^56^ and a distinct increase in FAD production. This reflects the enhanced mitochondrial oxidative metabolism,^57^ and this outcome is corroborated by the autofluorescence results.^58,59^ In MCF-7, a significant (*p* < 0.05) peak shift for NADH is observed (Figure 5C) at day 7 (470 nm) compared to days 3 and 14 (450 nm).

**Figure 5:**
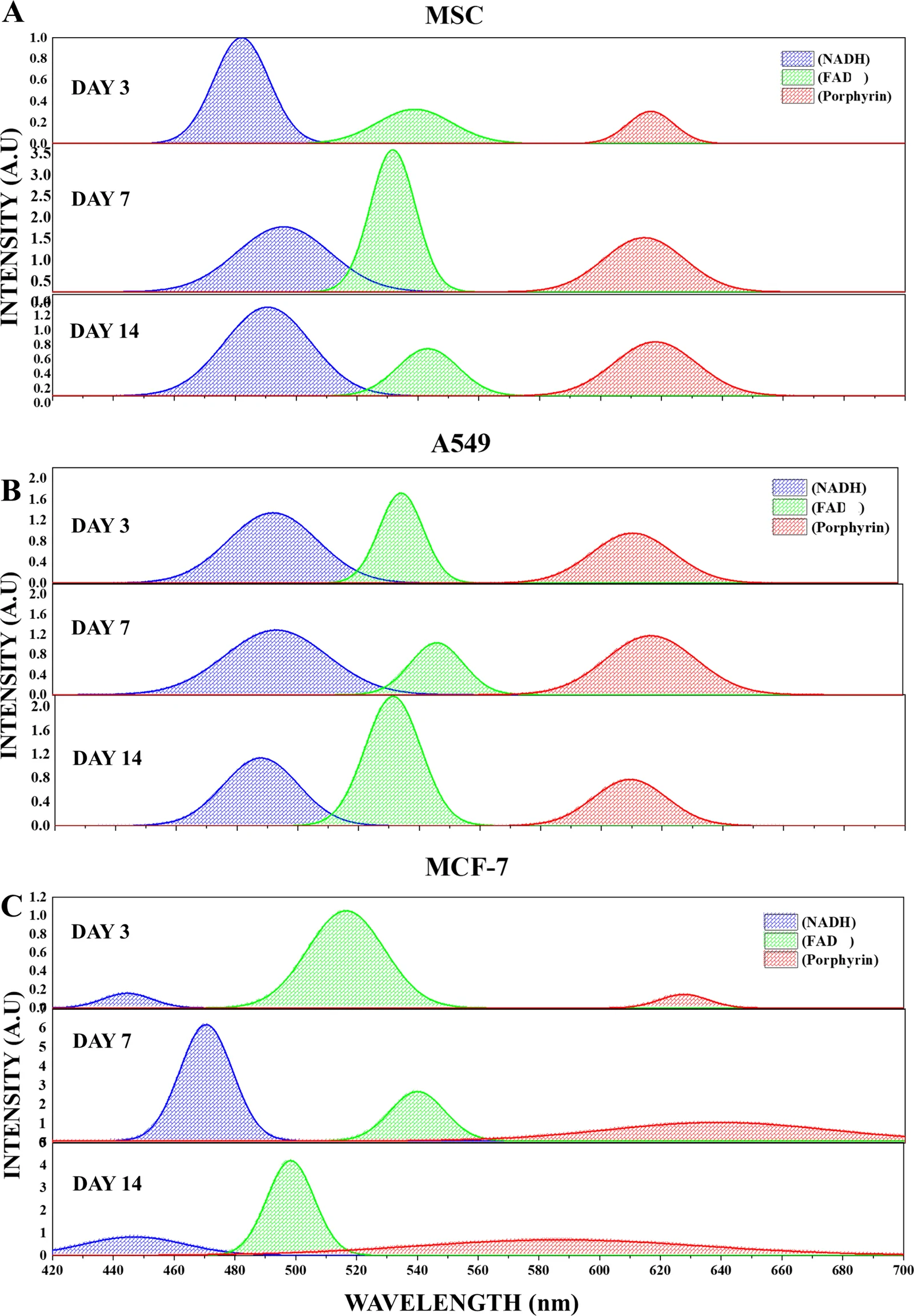
The graphical representation shows normalized spectral intensities of autofluorescence of (A) MSC, (B) A549, and (C) MCF-7 spheroids at 3, 7, and 14 days. Spheroids were excited at 405, 488, and 561 nm, and emissions were recorded at 440–495, 500–550, and 590–650 nm, respectively.

A549 spheroids show the highest accumulation of porphyrin (*p* < 0.05) on day 3 (Figure 4E). Porphyrin intensity increases significantly (*p* < 0.05) for all spheroids on day 7 as compared to day 3. Although all spheroids show lower intensity of porphyrin by day 14, a significant (*p* < 0.05) shift in the peak from 620 nm at day 3 to 640 nm at days 7 and 14 is observed in MCF-7, indicating altered heme-biosynthesis and dysregulated mitochondrial function.

Initially (on day 3), higher NADH and FAD accumulation is observed in A549, indicating an early metabolic hyperactivation.^48^ The MSC, on the other hand, maintains a metabolism with balanced glycolysis and mitochondrial activity.^55^ However, during the maturation stage (on day 7), the metabolic dynamics of MSCs shift more toward the TCA cycle and oxidative phosphorylation (OxPhos). At a later stage (day 14), both cancer spheroids maintain higher FAD and porphyrin accumulations, indicating mitochondrial dysfunction associated with cancer.^56,57^

The metabolic profile reveals distinct metabolic dynamics in MSC, A549, and MCF-7 spheroids at different maturation stages. Similar to morphological assessment, MSC spheroids show a controlled shift toward oxidative metabolism, with cancer spheroids exhibiting higher levels of NADH, FAD, and porphyrins, suggesting metabolic reprogramming and mitochondrial dysfunction.

### Electrical Dynamics Assessment of Spheroids

Impedance analysis is performed to evaluate the dielectric behavior of spheroids, associated with cellular viability, spheroid growth kinetics, and metabolic dynamics. Changes in impedance are closely associated with spheroid size, biomass accumulation, and membrane integrity. Electrical properties sensitively detect minute differences in spheroid structure and the spatial arrangement of cells across culture time.^29,37^

The frequency-dependent impedance spectra of spheroids derived from MSC, A549, and MCF-7 cells are represented in Figure 6A–C. On day 3, both A549 and MSC spheroids show comparable impedance, with values of 273 and 275 Ω at 10^3^ Hz and 173 and 171 Ω at 10^6^ Hz, respectively. The resemblance in electrical behavior is possibly due to the presence of stemness-associated traits in A549 and MSC. The MCF-7 spheroid consistently shows the lowest impedance, ranging from 136 to 250 Ω. On day 7, MSC spheroids exhibit the highest impedance amplitude (460–156 Ω), while the A549 spheroid shows lower amplitudes (431–146 Ω). In MCF-7 spheroids, a significant (*p* < 0.0001) reduction in impedance value is observed (250–133 Ω) on day 7. By day 14, the A549 spheroid exhibits the highest impedance amplitude (300 Ω–156 Ω), while MCF-7 and MSC spheroids cluster closer together with lower ranges (249–133 and 247–133 Ω, respectively).

**Figure 6:**
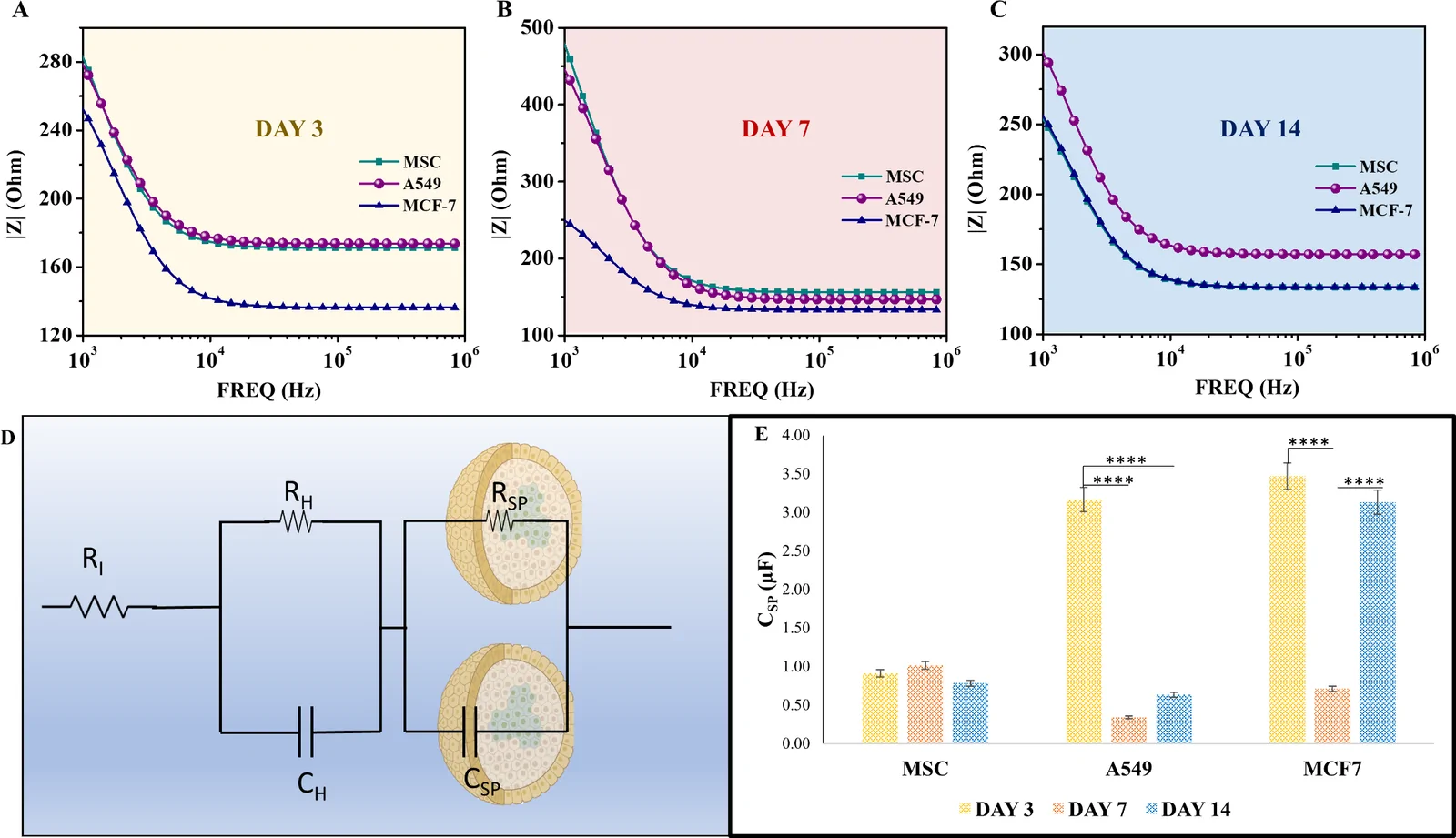
Bode plots of impedance [|*Z*|] vs frequency for MSC, A549, and MCF-7 spheroids cultured for (A) 3, (B) 7, and (C) 14 day. (D) The impedance data are fitted to the circuit model. *R*_H_ and *C*_H_ are the resistance and capacitance of the hydrogel in which spheroids are encapsulated. *R*_SP_ and *C*_SP_ are the resistance and capacitance present inside the proliferative (outermost) layer of the spheroids. (*E*) Graphical representation of the *C*_Sp_ (μF) of MSC, A549, and MCF-7 spheroids cultured for 3, 7, and 14 days. The presentation of the data (E) is in mean ± S.D, *n* = 3. A significant difference is set at ****p < 0.0001.

To interpret these findings, a three-component equivalent circuit is employed (Figure 6D). This model consists of an interfacial resistance (*R*_I_), accounting for the electrode–electrolyte interface at low frequencies; a hydrogel component (*R*_H_ and *C*_H_), representing the alginate–gelatin encapsulating medium, with *R*_H_ corresponding to the conductive pathways through the hydrogel and *C*_H_ representing its capacitive storage; and a spheroid component (*R*_SP_ and *C*_SP_), where *R*_SP_ represents the ionic resistivity of the spheroid bulk and *C*_SP_ reflects the capacitance of the proliferative outer layer. These elements are arranged so that *R*_H_ and *C*_H_ are in parallel, *R*_SP_ and *C*_SP_ are in parallel, and both are in series with *R*_I_. This configuration is consistent with established models of multicellular spheroids and tissue-like constructs, in which the dielectric dispersion arises from the serial current path through the interfacial layer, the hydrogel, and the spheroid.

The capacitance (*C*_SP_) derived from equivalent circuit fitting highlights distinct temporal dynamics. On day 3, MCF-7 and A549 spheroids exhibit significantly (*p* < 0.0001) higher capacitance values (3.47 and 3.17 μF, respectively) than MSC spheroids (0.91 μF). By day 7, capacitance decreases for MCF-7 spheroids (0.71 μF), although MSC shows a rebound to 1.01 μF. A549 spheroids, on the other hand, drop significantly (*p* < 0.0001) to 0.34 μF. By day 14, both MCF-7 and A549 spheroids show significant (*p* < 0.0001) recovery from day 7 (3.13 and 0.64 μF, respectively). MSC spheroids, however, show a reduction in capacitance to 0.78 μF as compared to day 7, suggesting tighter compaction and reduced dielectric response at later stages (Figure 6E).

Impedance-derived resistivity and capacitance probe distinct and spatially heterogeneous spheroid properties, where system resistivity reflects membrane integrity, cell–cell cohesion, and bulk ionic mobility within the spheroid–hydrogel construct, while capacitance predominantly arises from membrane polarization of the viable outer layers. At day 3, the comparable resistivity of A549 and MSC spheroids across frequencies indicates similar ionic confinement and regulated membrane barriers, suggesting the persistence of stemness-associated electrochemical organization in A549 at early stages. In contrast, MCF-7 spheroids consistently exhibit lower resistivity, consistent with leaky membranes, weakened junctional integrity, and enhanced ionic transport typical of differentiated malignant cells. By day 7, MSC spheroids show the highest resistivity, reflecting increased compaction and reduced conductive pathways during maturation, while A549 displays slightly lower values, indicative of emerging heterogeneity as stem-like regulation begins to diverge. The persistently low resistivity of MCF-7 at this stage points to disordered architecture and necrotic core formation, facilitating charge transport. By day 14, A549 spheroids exhibit the highest resistivity, consistent with aggressive growth, altered membrane polarization, and restricted ionic mobility, marking a shift toward a more malignant electrochemical state. In contrast, MSC and MCF-7 converge toward lower resistivity ranges due to stabilized compaction in MSC and sustained membrane leakiness in MCF-7, respectively, highlighting how resistivity sensitively captures cell-state-dependent structural and functional transitions.

The dynamic evolution of spheroid capacitance (CSP) provides insight into membrane polarization, proliferative activity, and the organization of viable outer layers, as capacitance reflects the ability of cell membranes to store charge and is governed by membrane surface area, lipid composition, intercellular junctions, and packing density. Accordingly, the elevated capacitance observed in cancer spheroids (MCF-7 and A549) at day 3 indicates highly active proliferative cells with enhanced membrane surface area and dielectric polarization, characteristic of rapidly dividing malignant cells, whereas MSC spheroids exhibit lower initial capacitance consistent with regulated membrane dynamics and tighter intercellular coupling. Day 7 emerges as a functional transition point, where capacitance trends diverge due to cell-state-specific responses rather than simple growth effects: MCF-7 and A549 spheroids show a pronounced capacitance drop associated with abnormal growth, increased structural entropy, and necrotic core formation that reduce effective membrane polarization, while MSC spheroids display a transient capacitance increase, suggesting adaptive membrane remodelling and metabolic activation within the engineered microenvironment. By day 14, capacitance patterns reflect spheroid maturation and lineage commitment, with MCF-7 spheroids showing strong capacitance recovery due to re-established proliferative outer layers despite internal necrosis, A549 spheroids exhibiting a moderate increase consistent with their heterogeneous phenotype, and MSC spheroids demonstrating reduced capacitance due to compaction, stabilized membrane organization, and diminished proliferative activity. Overall, capacitance emerges as a sensitive functional descriptor of spheroid state, capturing transitions between proliferation, stress adaptation, and maturation.

The impedance and capacitance analyses show the distinct dielectric signatures of spheroids, reflecting differences in membrane integrity and proliferative activity based on their origin. This study demonstrates that the electrical profiling can sensitively reflect the functional transitions and lineage-specific behaviors in normal and cancer spheroids.

Overall, MSC spheroids exhibit higher system resistivity than cancer spheroids, reflecting tighter membrane integrity, stronger cell–cell junctions, and more effective ionic confinement. These dielectric properties align with their compact architecture (Figure 3C) and smaller spheroid size (Figures 1C and 2B). Uniform autofluorescence further supports reduced metabolic heterogeneity and preserved cellular homeostasis.^60^ In contrast, cancer spheroids display reduced resistivity due to increased membrane permeability, disrupted junctional organization, and enhanced ionic mobility associated with malignant phenotypes. The dielectric instability corresponds with increased spheroid size (Figure 1C), elevated redox ratios (Figure 4D), and autofluorescence gradients. Together, these features indicate metabolic reprogramming that alters membrane polarization and ionic regulation.^61^

The strong temporal correspondence between resistivity, capacitance dynamics, spheroid volume, and redox ratio demonstrates that impedance-derived parameters sensitively capture the coupling between morphodynamics, metabolic state, and dielectric behavior. Notably, the convergence of resistive profiles between A549 and MSC spheroids at early time points highlights shared stemness-associated electrochemical traits, including regulated membrane polarization and intermediate ionic barriers. By day 14, A549 spheroids diverge toward a distinctly cancer-like profile, characterized by altered membrane polarization and reduced ionic regulation, underscoring a progressive shift from stem-like plasticity to malignant reprogramming. Collectively, the integrated impedance analysis not only discriminates normal and cancer spheroids based on their electrochemical signatures but also reveals the dynamic coexistence and temporal evolution of stem-like and malignant traits within cancer stem cell–enriched spheroids.

Integrated analysis of morphometric, viability, metabolic, cytoskeletal, and electrochemical parameters demonstrates that spheroid maturation is governed by the synchronized structural, bioenergetic, and dielectric remodelling. MSC spheroids exhibit controlled growth and metabolic transitions, whereas cancer spheroids exhibit rapid proliferation, altered metabolism, and distinct dielectric signatures. This multimodal framework links growth, metabolic, and electrochemical dynamics across spheroids of stem cells, cancer stem cells, and differentiated cancer cells, providing insights into cellular functional status and the cross-talk between regenerative and malignant pathways.

## Conclusions

Spheroids cultured in an ECM-mimetic hydrogel provide a powerful platform to study morphological, metabolic, and electrochemical dynamics across stem, cancer stem cell-enriched, and differentiated cancer populations. A549 spheroids display both stem-like and malignant traits, while day 7 marks a critical transition with abnormal growth and necrotic core formation, stabilizing by day 14. Integration of autofluorescence and impedance spectroscopy with imaging enables noninvasive tracking of redox status, dielectric response, and morpho-functional evolution. This multimodal approach advances understanding of tissue regeneration and cancer progression, while highlighting potential therapeutic targets. The platform holds translational potential for patient-specific drug screening, organoid-on-chip models, tissue engineering, and regenerative medicine applications.

## Supplementary Material



## Notes

Additional data analysis of autofluorescence intensities using Gaussian fit in Origin for normalization, and visual representation of the measurement setup for spheroid impedance (PDF)

## Abbreviations

A549: lung adenocarcinoma
MSC: mesenchymal stem cell
DMEM: Dulbecco modified Eagle medium
RPMI: Roswell Park Memorial Institute
3D model: three-dimensional model
FBS: fetal bovine serum
DAPI: 4′,6-diamidino-2-phenylindole
ECM: extracellular matrix
MCF-7: breast adenocarcinoma
SEM: scanning electron microscope.

## References

[1] Nayak P., Bentivoglio V., Varani M., Signore A. (2023). Three-dimensional in
vitro tumor spheroid models for evaluation of anticancer therapy:
recent updates. Cancers.

[2] Arora S., Singh S., Mittal A., Desai N., Khatri D. K., Gugulothu D., Lather V., Pandita D., Vora L. K. (2024). Spheroids
in cancer research: Recent advances and opportunities. Journal of Drug Delivery Science and Technology.

[3] Nath S., Devi G. R. (2016). Three-dimensional
culture systems in cancer research:
Focus on tumor spheroid model. Pharmacology
& therapeutics.

[4] Chiaradia I., Imaz-Rosshandler I., Nilges B. S., Boulanger J., Pellegrini L., Das R., Kashikar N. D., Lancaster M. A. (2023). Tissue
morphology influences the temporal program of human brain organoid
development. Cell Stem Cell.

[5] El
Harane S., Zidi B., El Harane N., Krause K.-H., Matthes T., Preynat-Seauve O. (2023). Cancer spheroids
and organoids as novel tools for research and therapy: state of the
art and challenges to guide precision medicine. Cells.

[6] Białkowska K., Komorowski P., Bryszewska M., Miłowska K. (2020). Spheroids
as a type of three-dimensional cell cultures—examples of methods
of preparation and the most important application. International journal of molecular sciences.

[7] Lee S.-Y., Koo I.-S., Hwang H. J., Lee D. W. (2023). In Vitro three-dimensional
(3D) cell culture tools for spheroid and organoid models. SLAS discovery.

[8] Habanjar O., Diab-Assaf M., Caldefie-Chezet F., Delort L. (2021). 3D cell culture systems:
tumor application, advantages, and disadvantages. International journal of molecular sciences.

[9] Aguilar
Cosme J. R., Gagui D. C., Bryant H. E., Claeyssens F. (2021). Morphological
response in cancer spheroids for screening photodynamic therapy parameters. Frontiers in molecular biosciences.

[10] Rodriguez-Gatica J. E., Iefremova V., Sokhranyaeva L., Yeung S. W. C. A., Breitkreuz Y., Brüstle O., Schwarz M. K., Kubitscheck U. (2022). Imaging three-dimensional
brain organoid architecture from meso-to nanoscale across development. Development.

[11] Caprio N. D., Burdick J. A. (2023). Engineered biomaterials
to guide spheroid formation,
function, and fabrication into 3D tissue constructs. Acta Biomater..

[48] Cheng G., Tse J., Jain R. K., Munn L. L. (2009). Micro-environmental mechanical stress
controls tumor spheroid size and morphology by suppressing proliferation
and inducing apoptosis in cancer cells. PLoS
one.

[13] Beck L. E., Lee J., Coté C., Dunagin M. C., Lukonin I., Salla N., Chang M. K., Hughes A. J., Mornin J. D., Gartner Z. J., Liberali P., Raj A. (2022). Systematically quantifying morphological
features reveals constraints on organoid phenotypes. Cell systems.

[14] Gjorevski N., Nikolaev M., Brown T. E., Mitrofanova O., Brandenberg N., DelRio F. W., Yavitt F. M., Liberali P., Anseth K. S., Lutolf M. P. (2022). Tissue geometry drives deterministic
organoid patterning. Science.

[15] Chen H., Jiang B., Shamul J. G., He X. (2021). Image entropy-based
label-free functional characterization of human induced pluripotent
stem cell-derived 3D cardiac spheroids. Biosens.
Bioelectron..

[16] Liu Y., Jiao Y., Fan Q., Li X., Liu Z., Qin D., Hu J., Liu L., Shuai J., Li Z. (2024). Morphological
entropy encodes cellular migration strategies on multiple length scales. npj Syst. Biol. App..

[17] Srivastava, V. ; Hu, J. L. ; Garbe, J. C. ; Veytsman, B. ; Shalabi, S. F. ; Yllanes, D. ; Thomson, M. ; LaBarge, M. A. ; Huber, G. ; Gartner, Z. J. Configurational entropy is an intrinsic driver of tissue structural heterogeneity. BioRxiv 2023.

[18] Kim S., Kim E. M., Yamamoto M., Park H., Shin H. (2020). Engineering
multi-cellular spheroids for tissue engineering and regenerative medicine. Adv. Healthcare Mater..

[19] Abbas Z. N., Al-Saffar A. Z., Jasim S. M., Sulaiman G. M. (2023). Comparative analysis
between 2D and 3D colorectal cancer culture models for insights into
cellular morphological and transcriptomic variations. Sci. Rep..

[20] Kolenc O. I., Quinn K. P. (2019). Evaluating cell
metabolism through autofluorescence
imaging of NAD (P) H and FAD. Antioxidants &
redox signaling.

[21] Dornhof J., Kieninger J., Muralidharan H., Maurer J., Urban G. A., Weltin A. (2022). Microfluidic organ-on-chip system for multi-analyte
monitoring of metabolites in 3D cell cultures. Lab Chip.

[22] Kilin V., Mas C., Constant S., Wolf J. P., Bonacina L. (2017). Health state dependent
multiphoton induced autofluorescence in human 3D in vitro lung cancer
model. Sci. Rep..

[23] Hu, L. ; Walsh, A. J. Identifying cancer cell metabolic states in autofluorescence lifetime images with machine learning. In Label-free Biomedical Imaging and Sensing (LBIS) 2023; SPIE: 2023 Vol. 12391, pp 11–15.

[24] Palmer S., Litvinova K., Dunaev A., Fleming S., McGloin D., Nabi G. (2016). Changes in autofluorescence based
organoid model of muscle invasive
urinary bladder cancer. Biomedical optics express.

[25] Gharekhan A. H., Arora S., Oza A. N., Sureshkumar M. B., Pradhan A., Panigrahi P. K. (2011). Distinguishing autofluorescence of
normal, benign, and cancerous breast tissues through wavelet domain
correlation studies. J. Biomed. Optics.

[26] Yuan Y., Yan Z., Miao J., Cai R., Zhang M., Wang Y., Wang L., Dang W., Wang D., Xiang D., Wang Y., Zhang P., Cui Y., Bian X., Ma Q. (2019). Autofluorescence of NADH is a new
biomarker for sorting and characterizing
cancer stem cells in human glioma. Stem Cell
Res. Ther..

[27] Koo K.-M., Kim C.-D., Kim T.-H. (2024). Recent
advances in electrochemical
detection of cell energy metabolism. Biosensors.

[28] Venkatesh H. S. (2023). Targeting
electrochemical communication between neurons and cancer. Sci. Transl. Med..

[29] Abasi S., Aggas J. R., Garayar-Leyva G. G., Walther B. K., Guiseppi-Elie A. (2022). Bioelectrical
impedance spectroscopy for monitoring mammalian cells and tissues
under different frequency domains: A review. ACS measurement science au.

[30] Ahn J., Jung K. B., Kwon O., Choi M.-S., Ahn J.-H., Han H.-Y., Jung C.-R., Yoon S., Son M.-Y., Oh J.-H. (2021). Impedance Measurement
System for Assessing the Barrier Integrity
of Three-Dimensional Human Intestinal Organoids. Anal. Chem..

[31] Akilavasan J., Marlow F. (2020). Electrochemical impedance spectroscopy
at redox-type
liquid| liquid interfaces: the capacitance lag. J. Phys. Chem. C.

[32] Gerasimenko T., Nikulin S., Zakharova G., Poloznikov A., Petrov V., Baranova A., Tonevitsky A. (2020). Impedance
spectroscopy as a tool for monitoring performance in 3D models of
epithelial tissues. Front. Bioeng. Biotechnol..

[33] Adhikary, P. ; Paul, R. ; Zhao, Y. ; Islam, K. ; Qin, X. ; Liu, Y. Label Free in Situ Characterization of 3d Tumor Spheroids by Microfluidic-Based Bioimpedance Spectroscopy. Available at SSRN 5200814.

[34] Marino A., Ziaja K., Lefevre M. C., Ceccarelli M. C., Battaglini M., Filippeschi C., Ciofani G. (2025). High-throughput impedance
monitoring in 3D tumor cultures: a multiplex, microfluidic-free platform
for drug screening. Lab Chip.

[35] Schmid Y. R., Burgel S. C., Misun P. M., Hierlemann A., Frey O. (2016). Electrical impedance spectroscopy for microtissue spheroid analysis
in hanging-drop networks. ACS sensors.

[36] Amaral R. L. F., Miranda M., Marcato P. D., Swiech K. (2017). Comparative analysis
of 3D bladder tumor spheroids obtained by forced floating and hanging
drop methods for drug screening. Front. Physiol..

[37] Wu H., Yang Y., Bagnaninchi P. O., Jia J. (2018). Electrical impedance
tomography for real-time and label-free cellular viability assays
of 3D tumour spheroids. Analyst.

[38] Karakas D., Sigirli S. (2025). Impact of different
3D culture environments on spheroid
morphology, drug response, and invasion in pancreatic cancer cells. BMC Res. Notes.

[39] Bess S. N., Smart G. K., Igoe M. J., Muldoon T. J. (2024). Scaffold-free development
of multicellular tumor spheroids with spatial characterization of
structure and metabolic radial profiles. In
vitro models.

[40] Thakuri P. S., Gupta M., Plaster M., Tavana H. (2019). Quantitative
size-based
analysis of tumor spheroids and responses to therapeutics. ASSAY and Drug Development Technologies.

[41] Parigoris E., Lee S., Mertz D., Turner M., Liu A. Y., Sentosa J., Djomehri S., Chang H. C., Luker K., Luker G., Kleer C. G., Takayama S. (2021). Cancer Cell Invasion of Mammary Organoids
with Basal-In Phenotype. Adv. Healthcare Mater..

[42] Malik A. A., Nguyen K. C., Nardini J. T., Krona C. C., Flores K. B., Nelander S. (2025). Mathematical modeling of multicellular
tumor spheroids
quantifies inter-patient and intra-tumor heterogeneity. npj Syst. Biol. App..

[43] Carrasco-Mantis A., Randelovic T., Castro-Abril H., Ochoa I., Doblaré M., Sanz-Herrera J. A. (2023). A mechanobiological model for tumor spheroid evolution
with application to glioblastoma: a continuum multiphysics approach. Computers in Biology and Medicine.

[44] Murphy K. C., Hung B. P., Browne-Bourne S., Zhou D., Yeung J., Genetos D. C., Leach J. K. (2017). Measurement
of oxygen tension within
mesenchymal stem cell spheroids. J. R. Soc.
Interface.

[45] Lugand L., Mestrallet G., Laboureur R., Dumont C., Bouhidel F., Djouadou M., Masson-Lecomte A., Desgrandchamps F., Culine S., Carosella E. D., Rouas-Freiss N., LeMaoult J. (2022). Methods for establishing a renal
cell carcinoma tumor
spheroid model with immune infiltration for immunotherapeutic studies. Front. Oncol..

[46] Min S., Choe C., Roh S. (2021). AQP3 increases
intercellular cohesion
in NSCLC A549 cell spheroids through exploratory cell protrusions. International journal of molecular sciences.

[47] Zhu S., Yin J., Lu X., Jiang D., Chen R., Cui K., He W., Huang N., Xu G. (2025). Influence of experimental variables
on spheroid attributes. Sci. Rep..

[49] Yildiz-Ozturk E., Saglam-Metiner P., Yesil-Celiktas O. (2021). Lung carcinoma
spheroids embedded
in a microfluidic platform. Cytotechnology.

[50] Weems A. D., Welf E. S., Driscoll M. K., Zhou F. Y., Mazloom-Farsibaf H., Chang B. J., Murali V. S., Gihana G. M., Weiss B. G., Chi J., Rajendran D., Dean K. M., Fiolka R., Danuser G. (2023). Blebs promote
cell survival by assembling oncogenic signalling hubs. Nature.

[51] Pulze L., Congiu T., Brevini T. A., Grimaldi A., Tettamanti G., D’antona P., Baranzini N., Acquati F., Ferraro F., de Eguileor M. (2020). MCF7 spheroid
development: New insight about spatio/temporal
arrangements of TNTs, amyloid fibrils, cell connections, and cellular
bridges. International journal of molecular
sciences.

[52] Barbosa M. A., Xavier C. P., Pereira R. F., Petrikaitė V., Vasconcelos M. H. (2022). 3D cell culture models as recapitulators of the tumor
microenvironment for the screening of anti-cancer drugs. Cancers.

[53] Mishra D. K., Miller R. A., Pence K. A., Kim M. P. (2018). Small cell and non
small cell lung cancer form metastasis on cellular 4D lung model. BMC cancer.

[54] Schilling K., Brown E., Zhang X. (2022). NAD (P) H
autofluorescence lifetime
imaging enables single cell analyses of cellular metabolism of osteoblasts
in vitro and in vivo via two-photon microscopy. Bone.

[55] Tee C. A., Roxby D. N., Othman R., Denslin V., Bhat K. S., Yang Z., Han J., Tucker-Kellogg L., Boyer L. A. (2024). Metabolic modulation to improve MSC
expansion and therapeutic
potential for articular cartilage repair. Stem
Cell Res. Ther..

[56] Walsh A. J., Cook R. S., Sanders M. E., Aurisicchio L., Ciliberto G., Arteaga C. L., Skala M. C. (2014). Quantitative
optical
imaging of primary tumor organoid metabolism predicts drug response
in breast cancer. Cancer research.

[57] Tomková V., Sandoval-Acuña C., Torrealba N., Truksa J. (2019). Mitochondrial fragmentation, elevated mitochondrial
superoxide and respiratory supercomplexes disassembly is connected
with the tamoxifen-resistant phenotype of breast cancer cells. Free Radical Biol. Med..

[58] Morrow C. S., Tweed K., Farhadova S., Walsh A. J., Lear B. P., Roopra A., Risgaard R. D., Klosa P. C., Arndt Z. P., Peterson E. R., Chi M. M., Harris A. G., Skala M. C., Moore D. L. (2024). Autofluorescence
is a biomarker of neural stem cell
activation state. Cell Stem Cell.

[59] Yan Y., Xing F., Cao J., Hu Y., Li L., Gao Z., Jia H., Miao K., Shao F., Deng C. X., Luo K. Q., Lee L. T. o., Liu T. M. (2023). Fluorescence intensity
and lifetime imaging of lipofuscin-like autofluorescence for label-free
predicting clinical drug response in cancer. Redox Biol..

[60] Ohori-Morita Y., Ashry A., Niibe K., Egusa H. (2025). Current perspectives
on the dynamic culture of mesenchymal stromal/stem cell spheroids. Stem Cells Transl. Med..

[61] Wang W., Liu Y., Huang X., Liang F., Luo H., Mao Z., Shi J., Wang L., Peng J., Chen Y. (2024). Diffusion-based culture
and real-time impedance monitoring of tumor spheroids in hydrogel
microwells of a suspended membrane under microfluidic conditions. Talanta.

