# Supplementary material for: Multimodal Biophysical Analysis of Morpho-Phenotypic and Morpho-Functional Dynamics in Normal and Cancer Spheroids within Engineered Microenvironments
Source: Chem Biomed Imaging. 2026 Apr 29;4(9):2010–22. doi: 10.1021/cbmi.5c00255 (PMC13625695; doi:10.1021/cbmi.5c00255)
Supplement: Supplementary file 1 [file im5c00255_si_001.pdf]

Supporting Information

Multimodal Biophysical Analysis of Morpho-Phenotypic and Morpho-Functional Dynamics in Normal and Cancer Spheroids within Engineered Microenvironments

Srijani Dey[1], Shreya Adhikary[1], Priyanka Das[2], Pallab Datta[2] , Subhadip Chakraborty[3,4*], Ananya Barui[1*]

[1]*Centre for Healthcare Science and Technology, Indian Institute of Engineering Science and Technology, Shibpur, Howrah-711103*

[2]*Department of Pharmaceutics, National Institute of Pharmaceutical Education and Research, Kolkata 700054, India*

[3]*Institute of Engineering and Management, Management House, D-1, Sector-V, Saltlake Electronics Complex, 700091, Kolkata, West Bengal, India*

[4]*University of Engineering and Management, New Town, University, Area, Plot no. III, B/5, New Town Rd, Action Area III, Newtown, 700160, Kolkata, West Bengal, India*

***Correspondence Authors**

E-mail addresses: ananya.pariksha@gmail.com; ananyabarui.chest@faculty.iiests.ac.in (Dr. Ananya Barui)**;** subhadip1310@gmail.com (Dr. Subhadip Chakraborty).

1. **Analysing the metabolic parameter using the Origin 8**: To assess the intrinsic autofluorescence, non-fluorescent labelled fixed spheroids were imaged at 10x magnification. The spheroids were excited using laser scanning confocal lasers (Leica Stellaris 5, Germany) at 405nm, 488 nm, 514nm, 561nm and 638nm. The signals were detected between 410-480nm, 490-510nm, 520-560nm, 565-620nm and 640-750nm. Laser power was optimized to 50%, and 13 scans were taken for each wavelength. Experiments were performed in triplicates. Normalisation of the data was done using NonLinear Curve Fit (model: Gaussian) and the equation:

$$y = y0 + A/(w \times \sqrt{(\pi/(4 \times \log_e 2))} \times \exp\left(-4 \times \log_e 2 \times (x - xc)^2 / w^2\right)$$

The calculation for the above-normalised data is provided below:

Table S1: The table for the calculation of autofluorescence intensities for MSC, A549, and MCF-7 spheroids at 3,7 and 14 days.

Gaussian fit for autofluorescence using Origin 8

MSC

Day 3		y0	y0	xc	xc	A	A	w	w	Statistics	Statistics	
		Value	Standard Error	Value	Standard Error	Value	Standard Error	Value	Standard Error	Reduced Chi-Sqr	Adj. R-Square	Intensitites
	NADH	0.00128	0.00108	482.0586	4665742.4	22.71453	3851357	21.34648	3150077	2.70E-06	0.99997	1.000924
	FAD	0.00128	0.00108	538.9229	1197852.7	9.95035	610844.7	28.89494	872730.7			0.324787
	Porphyrin	0.00128	0.00108	616.5039	7887732.7	5.80014	1.65E+07	17.94945	1.81E+07			0.304847
Day 7		y0	y0	xc	xc	A	A	w	w	Statistics	Statistics	
		Value	Standard Error	Value	Standard Error	Value	Standard Error	Value	Standard Error	Reduced Chi-Sqr	Adj. R-Square	Intensities
	NADH	0.00303	0.00137	495.8501	2919.3322	58.78929	10749.82	36.15853	1783.218	7.46E-06	0.99997	1.530439
	FAD	0.00303	0.00137	531.7698	66822.523	61.88824	1662172	17.42571	154341.6			3.339486
	Porphyrin	0.00303	0.00137	614.3341	6.41415	42.387	3.0436	31.27448	16.50612			1.27627
Day 14		y0	y0	xc	xc	A	A	w	w	Statistics	Statistics	
		Value	Standard Error	Value	Standard Error	Value	Standard Error	Value	Standard Error	Reduced Chi-Sqr	Adj. R-Square	Intensities
	NADH	0.00212	0.0015	490.6543	3458.0635	43.36683	8538.121	33.40175	2366.622	8.95E-06	0.99991	1.221829
	FAD	0.00212	0.0015	543.3003	73603.593	16.20643	39056.58	23.46802	55893.23			0.650872
	Porphyrin	0.00212	0.0015	617.9686	1.29891	25.30718	1.65737	32.03496	8.50877			0.744263

A549

Day 3		y0	y0	xc	xc	A	A	w	w	Statistics	Statistics	
		Value	Standard Error	Value	Standard Error	Value	Standard Error	Value	Standard Error	Reduced Chi-Sqr	Adj. R-Square	Intensities
	NADH	0.00355	0.00208	493.0262	3481.9316	49.68928	10258.26	34.95535	2272.972	1.30E-05	0.99992	1.338967
	FAD	0.00355	0.00208	535.4988	86507.077	31.20079	425198	17.14217	103910.4			1.713438
	Porphyrin	0.00355	0.00208	612.1837	23.20772	32.15417	3.3019	31.84681	44.92831			0.952054
Day 7		y0	y0	xc	xc	A	A	w	w	Statistics	Statistics	
		Value	Standard Error	Value	Standard Error	Value	Standard Error	Value	Standard Error	Reduced Chi-Sqr	Adj. R-Square	
	NADH	0.00548	0.00328	493.6771	638.29682	54.21378	1769.22	39.76833	462.9312	4.29E-05	0.99976	1.286159
	FAD	0.00548	0.00328	546.5378	100434.33	22.50283	14661.82	20.57783	84611.81			1.032799
	Porphyrin	0.00548	0.00328	617.0448	1.3403	43.34626	0.40217	34.85018	6.93503			1.173941
Day 14		y0	y0	xc	xc	A	A	w	w	Statistics	Statistics	
		Value	Standard Error	Value	Standard Error	Value	Standard Error	Value	Standard Error	Reduced Chi-Sqr	Adj. R-Square	
	NADH	0.00166	9.13E-04	487.9059	24171.71	35.06502	49354.77	29.02953	16201.03	3.34E-06	0.99998	1.136414
	FAD	0.00166	9.13E-04	531.3939	17004.265	49.78969	189877.2	21.55634	50030.37			2.171522
	Porphyrin	0.00166	9.13E-04	609.4571	309.31166	23.52363	66.17164	28.53229	402.6639			0.776185

MCF-7												
Day 3		y0	y0	xc	xc	A	A	w	w	Statistics	Statistics	
		Value	Standard Error	Value	Standard Error	Value	Standard Error	Value	Standard Error	Reduced Chi-Sqr	Adj. R-Square	Intensities
	NADH	0.00206	0.00209	444.5932	367302.25	3.24031	84180.01	19.24321	385115	1.31E-05	0.99984	0.160249
	FAD	0.00206	0.00209	516.5878	255.77232	33.23094	117.0484	29.68	335.0995			1.053892
	Porphyrin	0.00206	0.00209	627.718	2174.583	2.92871	832.0476	18.75041	2793.321			0.148795
Day 7		y0	y0	xc	xc	A	A	w	w	Statistics	Statistics	
		Value	Standard Error	Value	Standard Error	Value	Standard Error	Value	Standard Error	Reduced Chi-Sqr	Adj. R-Square	Intensities
	NADH	0.10807	0.07846	495.045	50451.152	117.1942	4271277	18.11758	224226.5	0.02047	0.88836	6.184851
	FAD	0.10807	0.07846	556.9969	1252409.5	51.60383	4.91E+07	18.8424	6730569			2.680914
	Porphyrin	0.10807	0.07846	645.171	11.14132	77.72863	28.77807	75.82711	28.92197			1.071066
Day 14		y0	y0	xc	xc	A	A	w	w	Statistics	Statistics	
		Value	Standard Error	Value	Standard Error	Value	Standard Error	Value	Standard Error	Reduced Chi-Sqr	Adj. R-Square	Intensities
	NADH	0.01979	0.04319	446.9093	--	33.72382	--	39.07744	--	0.00556	0.95036	0.830524
	FAD	0.01979	0.04319	498.3504	--	81.15988	--	18.24648	--			4.198383
	Porphyrin	0.01979	0.04319	586.8195	11.40974	79.64599	21.37151	109.7261	27.14047			0.701691

2. Schematic Representation of the setup used for measuring impedance.

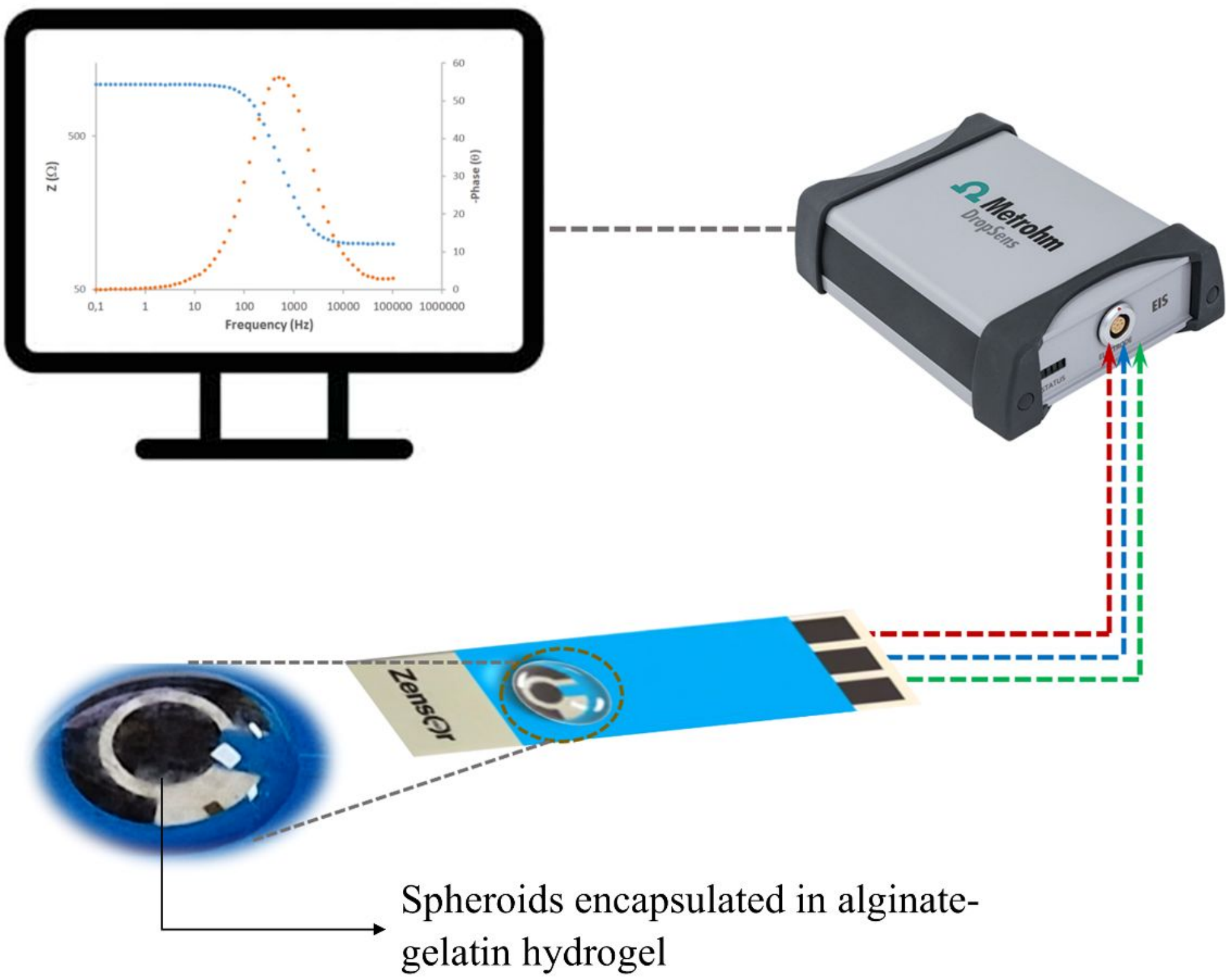

Figure S1: The schematic representation of the setup for measurement of impedance of spheroids. The magnified image shows a drop of buffer containing spheroids encapsulated in the alginate-gelatin hydrogel droplet.
